# Diaqua­bis(tetra­zolo[1,5-*a*]pyridine-8-carboxyl­ato-κ^2^ *N* ^1^,*O*)manganese(II) dihydrate

**DOI:** 10.1107/S1600536809023253

**Published:** 2009-06-24

**Authors:** Jian-De Zhao

**Affiliations:** a School of Chemistry and Chemical Engineering, Tianjin University of Technology, Tianjin 300191, People’s Republic of China

## Abstract

In the title compound, [Mn(C_6_H_3_N_4_O_2_)_2_(H_2_O)_2_]·2H_2_O, the Mn^II^ atom is located on a twofold rotation axis and is octa­hedrally coordinated by the N and O atoms of the chelating tetra­zolo[1,5-*a*]pyridine-8-carboxyl­ate anions and the O atoms of two water mol­ecules. Hydrogen bonds of the O—H⋯O and O—H⋯N types lead to the formation of layers parallel to (100).

## Related literature

For background to coordination compounds, see: Kulynych & Shimizu (2002 ▶); Liu *et al.* (2001 ▶); Xue & Liu (2009 ▶). 
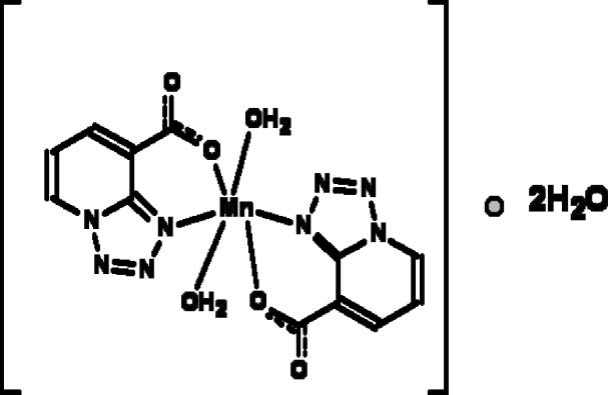


## Experimental

### 

#### Crystal data

[Mn(C_6_H_3_N_4_O_2_)_2_(H_2_O)_2_]·2H_2_O
*M* *_r_* = 453.25
Orthorhombic, 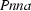

*a* = 19.041 (4) Å
*b* = 11.694 (2) Å
*c* = 7.5371 (15) Å
*V* = 1678.3 (6) Å^3^
*Z* = 4
Mo *K*α radiation
μ = 0.85 mm^−1^
*T* = 293 K
0.5 × 0.5 × 0.5 mm

#### Data collection

Rigaku SCXmini diffractometer
Absorption correction: multi-scan (*ABSCOR*; Higashi, 1995 ▶) *T*_min_ = 0.60, *T*_max_ = 0.662
16422 measured reflections
1925 independent reflections
1755 reflections with *I* > 2σ(*I*)
*R*_int_ = 0.029

#### Refinement

*R*[*F* ^2^ > 2σ(*F* ^2^)] = 0.032
*wR*(*F* ^2^) = 0.081
*S* = 1.20
1925 reflections
132 parameters
H-atom parameters constrained
Δρ_max_ = 0.26 e Å^−3^
Δρ_min_ = −0.34 e Å^−3^

### 

Data collection: *SCXmini* (Rigaku, 2006 ▶); cell refinement: *PROCESS-AUTO* (Rigaku, 1998 ▶); data reduction: *PROCESS-AUTO*; program(s) used to solve structure: *SHELXS97* (Sheldrick, 2008 ▶); program(s) used to refine structure: *SHELXL97* (Sheldrick, 2008 ▶); molecular graphics: *ORTEPIII* (Burnett & Johnson, 1996 ▶), *ORTEP-3 for Windows* (Farrugia, 1997 ▶) and *PLATON* (Spek, 2009 ▶); software used to prepare material for publication: *SHELXTL* (Sheldrick, 2008 ▶).

## Supplementary Material

Crystal structure: contains datablocks global, I. DOI: 10.1107/S1600536809023253/dn2463sup1.cif

Structure factors: contains datablocks I. DOI: 10.1107/S1600536809023253/dn2463Isup2.hkl

Additional supplementary materials:  crystallographic information; 3D view; checkCIF report

## Figures and Tables

**Table 1:** Hydrogen-bond geometry (Å, °)

*D*—H⋯*A*	*D*—H	H⋯*A*	*D*⋯*A*	*D*—H⋯*A*
O1*W*—H11⋯O2^i^	0.85	1.93	2.7644 (17)	166
O1*W*—H12⋯O2*W*^ii^	0.85	1.91	2.7538 (19)	171
O2*W*—H21⋯O1	0.89	1.95	2.8287 (18)	172
O2*W*—H22⋯N2^iii^	0.76	2.25	3.003 (2)	169

Symmetry codes: (i) 
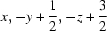
; (ii) 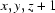; (iii) 
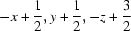
.

**Fig. 1.**
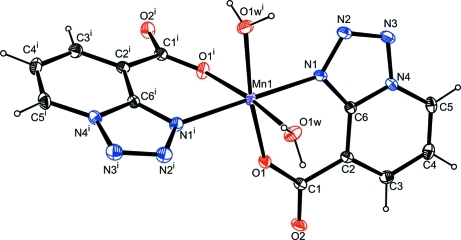
A view of the title compound showing the coordination of Mn atom with the atom-labelling scheme. Ellipsoids are drawn at the 30% probability level. H atoms and the solvate water molecule have been omitted for clarity. [ Symmetry codes: (i) -x+1/2, -y, z]

**Fig. 2.**
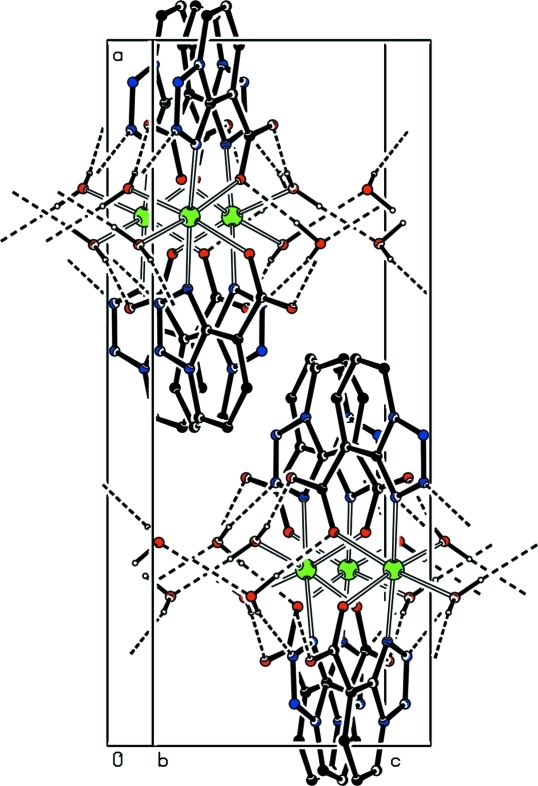
Partial packing view showing the formation of layers parallel to the (1 0 0) plane. H atoms not involved in hydrogen bondings have been omitted for clarity. H bonds are shown as dashed lines.

## supplementary crystallographic information

### Comment

Coordination complexes have attracted great attention in recent years. (Kulynych & Shimizu, 2002). Polydentate ligand have some heteroatom can coordinated to metal in different ways, and can form Hydrogen bonds between to give supermolecule net(Liu *et al.*, 2001). The tetrazolo(1,5-*a*)pyridine-8-carboxylate have multi-coordinated position and may behavs as a polydentate ligand. The related maganese structure with two water molecules as solvent has been recently reported (Xue & Liu, 2009).

In the title compound, the manganese atom is located on a two fold axis and octahedrally coordinated by two water molecules and two bidentate N,O tetrazolo(1,5-*a*)pyridine-8-carboxylate,(Fig. 1).

Each tetrazolo(1,5-*a*) pyridine-8-carboxylate chelates to one manganese atom. One type of water coordinates to the manganese atom whereas the other acts as lattice water. A two dimensional supramolecular network parallel to the (1 0 0) plane, is formed by the hydrogen bond interactions between the water molecules and the nitrogen of the tetrazolo(1,5-*a*)pyridine-8-carboxylate ligands (Table 1, Fig. 2).

The structure is closely related to the dihydrate complex (Xue & Liu, 2009), the only difference being the occurence of two solvate water molecules in the previous structure.

### Experimental

A mixture of manganeset(II)nitrate and sodium azide (1 mmol), 2-chloronicotinic acid(0.5 mmol), in 10 ml of water was sealed in a Teflon-lined stainless-steel Parr bomb that was heated at 363 K for 48 h. Red crystals of the title complex were collected after the bomb was allowed to cool to room temperature.Yield 20% based on manganese(II). Caution: Azides may be explosive. Although we have met no problems in this work, only a small amount of them should be prepared and handled with great caution.

### Refinement

All H atoms attached to C atoms were fixed geometrically and treated as riding with C—H = 0.93 Å and U_iso_(H) = 1.2U_eq_(C). H atoms of water molecule were located in difference Fourier maps and included in the subsequent refinement using restraints (O-H= 0.85 (1)Å and H···H= 1.39 (2)Å) with U_iso_(H) = 1.5U_eq_(O). In the last stage of refinement they were treated as riding on their parent O atoms.

### Crystal data

**Table:**

[Mn(C_6_H_3_N_4_O_2_)_2_(H_2_O)_2_]·2H_2_O	*F*(000) = 924
*M**_r_* = 453.25	*D*_x_ = 1.794 Mg m^−^^3^
Orthorhombic, *P**n**n**a*	Mo *K*α radiation, λ = 0.71073 Å
Hall symbol: -P 2a 2bc	Cell parameters from 15650 reflections
*a* = 19.041 (4) Å	θ = 3.2–27.9°
*b* = 11.694 (2) Å	µ = 0.85 mm^−^^1^
*c* = 7.5371 (15) Å	*T* = 293 K
*V* = 1678.3 (6) Å^3^	Block, yellow
*Z* = 4	0.5 × 0.5 × 0.5 mm

### Data collection

**Table:**

Rigaku SCXmini diffractometer	1925 independent reflections
Radiation source: fine-focus sealed tube	1755 reflections with *I* > 2σ(*I*)
graphite	*R*_int_ = 0.029
ω scans	θ_max_ = 27.5°, θ_min_ = 3.2°
Absorption correction: multi-scan (*ABSCOR*; Higashi, 1995)	*h* = −24→24
*T*_min_ = 0.60, *T*_max_ = 0.662	*k* = −15→15
16422 measured reflections	*l* = −9→9

### Refinement

**Table:**

Refinement on *F*^2^	Primary atom site location: structure-invariant direct methods
Least-squares matrix: full	Secondary atom site location: difference Fourier map
*R*[*F*^2^ > 2σ(*F*^2^)] = 0.032	Hydrogen site location: inferred from neighbouring sites
*wR*(*F*^2^) = 0.081	H-atom parameters constrained
*S* = 1.20	*w* = 1/[σ^2^(*F*_o_^2^) + (0.0386*P*)^2^ + 0.6168*P*] where *P* = (*F*_o_^2^ + 2*F*_c_^2^)/3
1925 reflections	(Δ/σ)_max_ = 0.001
132 parameters	Δρ_max_ = 0.26 e Å^−^^3^
0 restraints	Δρ_min_ = −0.34 e Å^−^^3^

### Special details

**Table:**

Geometry. All esds (except the esd in the dihedral angle between two l.s. planes) are estimated using the full covariance matrix. The cell esds are taken into account individually in the estimation of esds in distances, angles and torsion angles; correlations between esds in cell parameters are only used when they are defined by crystal symmetry. An approximate (isotropic) treatment of cell esds is used for estimating esds involving l.s. planes.
Refinement. Refinement of *F*^2^ against ALL reflections. The weighted *R*-factor *wR* and goodness of fit *S* are based on *F*^2^, conventional *R*-factors *R* are based on *F*, with *F* set to zero for negative *F*^2^. The threshold expression of *F*^2^ > σ(*F*^2^) is used only for calculating *R*-factors(gt) *etc*. and is not relevant to the choice of reflections for refinement. *R*-factors based on *F*^2^ are statistically about twice as large as those based on *F*, and *R*- factors based on ALL data will be even larger.

### Fractional atomic coordinates and isotropic or equivalent isotropic displacement parameters (Å^2^)

**Table:**

	*x*	*y*	*z*	*U*_iso_*/*U*_eq_	
Mn1	0.2500	0.0000	0.86700 (4)	0.01925 (12)	
O1	0.19724 (6)	0.09901 (10)	0.66846 (16)	0.0263 (3)	
O1W	0.21023 (6)	0.11467 (10)	1.06874 (17)	0.0294 (3)	
H11	0.1888	0.1746	1.0359	0.044*	
H12	0.2351	0.1270	1.1599	0.044*	
O2	0.12016 (6)	0.21215 (10)	0.53402 (17)	0.0306 (3)	
N1	0.14757 (7)	−0.09265 (11)	0.85974 (18)	0.0233 (3)	
N2	0.12692 (8)	−0.18779 (12)	0.9450 (2)	0.0279 (3)	
N3	0.05962 (8)	−0.20081 (12)	0.9411 (2)	0.0285 (3)	
N4	0.03454 (7)	−0.10924 (11)	0.84998 (17)	0.0212 (3)	
C1	0.13563 (9)	0.13028 (13)	0.6301 (2)	0.0201 (3)	
C2	0.07619 (8)	0.05909 (13)	0.7039 (2)	0.0194 (3)	
C3	0.00732 (9)	0.08539 (14)	0.6756 (2)	0.0244 (3)	
H3	−0.0036	0.1528	0.6161	0.029*	
C4	−0.04836 (9)	0.01396 (14)	0.7335 (2)	0.0276 (4)	
H4	−0.0946	0.0355	0.7122	0.033*	
C5	−0.03481 (9)	−0.08466 (15)	0.8190 (2)	0.0261 (4)	
H5	−0.0706	−0.1336	0.8551	0.031*	
C6	0.08933 (8)	−0.04294 (13)	0.7994 (2)	0.0185 (3)	
O2W	0.28824 (7)	0.12927 (11)	0.37555 (17)	0.0354 (3)	
H21	0.2590	0.1132	0.4635	0.053*	
H22	0.3103	0.1791	0.4079	0.053*	

### Atomic displacement parameters (Å^2^)

**Table:**

	*U*^11^	*U*^22^	*U*^33^	*U*^12^	*U*^13^	*U*^23^
Mn1	0.01548 (18)	0.02138 (19)	0.02089 (19)	0.00086 (12)	0.000	0.000
O1	0.0199 (6)	0.0302 (6)	0.0287 (6)	−0.0007 (5)	−0.0003 (5)	0.0108 (5)
O1W	0.0325 (7)	0.0280 (6)	0.0277 (6)	0.0091 (5)	−0.0033 (5)	−0.0048 (5)
O2	0.0297 (6)	0.0260 (6)	0.0362 (7)	−0.0035 (5)	−0.0053 (6)	0.0141 (5)
N1	0.0222 (7)	0.0181 (6)	0.0295 (7)	−0.0005 (5)	−0.0016 (5)	0.0066 (5)
N2	0.0295 (7)	0.0215 (7)	0.0326 (8)	−0.0017 (6)	0.0003 (6)	0.0083 (6)
N3	0.0312 (8)	0.0219 (7)	0.0324 (8)	−0.0038 (6)	0.0002 (6)	0.0079 (6)
N4	0.0215 (7)	0.0194 (6)	0.0226 (7)	−0.0044 (5)	0.0011 (5)	0.0018 (5)
C1	0.0230 (8)	0.0190 (7)	0.0185 (7)	−0.0021 (6)	−0.0002 (6)	0.0016 (6)
C2	0.0224 (8)	0.0175 (7)	0.0183 (7)	−0.0011 (6)	0.0004 (6)	0.0007 (6)
C3	0.0248 (8)	0.0245 (8)	0.0239 (8)	0.0018 (6)	−0.0025 (6)	0.0016 (6)
C4	0.0171 (7)	0.0369 (9)	0.0289 (9)	0.0015 (7)	−0.0009 (7)	−0.0018 (7)
C5	0.0178 (8)	0.0326 (9)	0.0279 (8)	−0.0068 (7)	0.0027 (7)	−0.0021 (7)
C6	0.0177 (7)	0.0187 (7)	0.0190 (7)	−0.0023 (6)	0.0001 (6)	−0.0003 (6)
O2W	0.0383 (8)	0.0386 (7)	0.0293 (7)	−0.0081 (6)	0.0032 (5)	−0.0043 (5)

### Geometric parameters (Å, °)

**Table:**

Mn1—O1	2.1422 (12)	N3—N4	1.3588 (19)
Mn1—O1^i^	2.1422 (12)	N4—C6	1.3546 (19)
Mn1—O1W	2.1642 (12)	N4—C5	1.372 (2)
Mn1—O1W^i^	2.1642 (12)	C1—C2	1.511 (2)
Mn1—N1	2.2317 (14)	C2—C3	1.364 (2)
Mn1—N1^i^	2.2317 (14)	C2—C6	1.416 (2)
O1—C1	1.262 (2)	C3—C4	1.418 (2)
O1W—H11	0.8477	C3—H3	0.9300
O1W—H12	0.8471	C4—C5	1.346 (2)
O2—C1	1.2362 (19)	C4—H4	0.9300
N1—C6	1.332 (2)	C5—H5	0.9300
N1—N2	1.3438 (19)	O2W—H21	0.8853
N2—N3	1.291 (2)	O2W—H22	0.7585
			
O1—Mn1—O1^i^	91.38 (7)	N2—N3—N4	105.51 (12)
O1—Mn1—O1W	89.53 (5)	C6—N4—N3	108.82 (13)
O1^i^—Mn1—O1W	171.80 (5)	C6—N4—C5	125.01 (14)
O1—Mn1—O1W^i^	171.80 (5)	N3—N4—C5	126.13 (14)
O1^i^—Mn1—O1W^i^	89.53 (5)	O2—C1—O1	125.44 (15)
O1W—Mn1—O1W^i^	90.73 (7)	O2—C1—C2	117.64 (14)
O1—Mn1—N1	80.53 (5)	O1—C1—C2	116.89 (13)
O1^i^—Mn1—N1	97.49 (5)	C3—C2—C6	116.08 (14)
O1W—Mn1—N1	90.70 (5)	C3—C2—C1	122.57 (14)
O1W^i^—Mn1—N1	91.27 (5)	C6—C2—C1	121.27 (14)
O1—Mn1—N1^i^	97.49 (5)	C2—C3—C4	122.53 (15)
O1^i^—Mn1—N1^i^	80.53 (5)	C2—C3—H3	118.7
O1W—Mn1—N1^i^	91.27 (5)	C4—C3—H3	118.7
O1W^i^—Mn1—N1^i^	90.70 (5)	C5—C4—C3	120.56 (16)
N1—Mn1—N1^i^	177.19 (7)	C5—C4—H4	119.7
C1—O1—Mn1	138.78 (10)	C3—C4—H4	119.7
Mn1—O1W—H11	118.4	C4—C5—N4	116.47 (15)
Mn1—O1W—H12	118.7	C4—C5—H5	121.8
H11—O1W—H12	111.4	N4—C5—H5	121.8
C6—N1—N2	106.31 (13)	N1—C6—N4	107.18 (13)
C6—N1—Mn1	121.61 (10)	N1—C6—C2	133.50 (14)
N2—N1—Mn1	130.24 (11)	N4—C6—C2	119.29 (14)
N3—N2—N1	112.16 (13)	H21—O2W—H22	105.7

Symmetry codes: (i) −*x*+1/2, −*y*, *z*.

### Hydrogen-bond geometry (Å, °)

**Table:**

*D*—H···*A*	*D*—H	H···*A*	*D*···*A*	*D*—H···*A*
O1W—H11···O2^ii^	0.85	1.93	2.7644 (17)	166
O1W—H12···O2W^iii^	0.85	1.91	2.7538 (19)	171
O2W—H21···O1	0.89	1.95	2.8287 (18)	172
O2W—H22···N2^iv^	0.76	2.25	3.003 (2)	169

Symmetry codes: (ii) *x*, −*y*+1/2, −*z*+3/2; (iii) *x*, *y*, *z*+1; (iv) −*x*+1/2, *y*+1/2, −*z*+3/2.
